# Antagonism of Histamine H3 receptors Alleviates Pentylenetetrazole-Induced Kindling and Associated Memory Deficits by Mitigating Oxidative Stress, Central Neurotransmitters, and c-Fos Protein Expression in Rats

**DOI:** 10.3390/molecules25071575

**Published:** 2020-03-30

**Authors:** Alaa Alachkar, Sheikh Azimullah, Mohamed Lotfy, Ernest Adeghate, Shreesh K. Ojha, Rami Beiram, Dorota Łażewska, Katarzyna Kieć-Kononowicz, Bassem Sadek

**Affiliations:** 1 Department of Pharmacology & Therapeutics, College of Medicine and Health Sciences, United Arab Emirates University, Al Ain 17666, UAE; 201590025@uaeu.ac.ae (A.A.); azim.sheikh@uaeu.ac.ae (S.A.); shreeshojha@uaeu.ac.ae (S.K.O.); rbeiram@uaeu.ac.ae (R.B.); 2 Department of Biology, College of Science, United Arab Emirates University, Al Ain 17666, UAE; m.lotfy@uaeu.ac.ae; 3 Department of Anatomy, College of Medicine and Health Sciences, United Arab Emirates University, Al Ain 17666, UAE; eadeghate@uaeu.ac.ae; 4 Jagiellonian University Medical College, Faculty of Pharmacy, Department of Technology and Biotechnology of Drugs, Medyczna 9 St., 30-688 Kraków, Poland; dlazewska@cm-uj.krakow.pl (D.Ł.); mfkonono@cyf-kr.edu.pl (K.K.-K.)

**Keywords:** histamine H3 receptor, antagonist, PTZ-kindling, memory impairment, neuroprotection, oxidative stress, AChE activity, c-Fos protein expression, rats

## Abstract

Histamine H3 receptors (H3Rs) are involved in several neuropsychiatric diseases including epilepsy. Therefore, the effects of H3R antagonist E177 (5 and 10 mg/kg, intraperitoneal (i.p.)) were evaluated on the course of kindling development, kindling-induced memory deficit, oxidative stress levels (glutathione (GSH), malondialdehyde (MDA), catalase (CAT), and superoxide dismutase (SOD)), various brain neurotransmitters (histamine (HA), acetylcholine (ACh), γ-aminobutyric acid (GABA)), and glutamate (GLU), acetylcholine esterase (AChE) activity, and c-Fos protein expression in pentylenetetrazole (PTZ, 40 mg/kg) kindled rats. E177 (5 and 10 mg/kg, i.p.) significantly decreased seizure score, increased step-through latency (STL) time in inhibitory avoidance paradigm, and decreased transfer latency time (TLT) in elevated plus maze (all *P* < 0.05). Moreover, E177 mitigated oxidative stress by significantly increasing GSH, CAT, and SOD, and decreasing the abnormal level of MDA (all *P* < 0.05). Furthermore, E177 attenuated elevated levels of hippocampal AChE, GLU, and c-Fos protein expression, whereas the decreased hippocampal levels of HA and ACh were modulated in PTZ-kindled animals (all *P* < 0.05). The findings suggest the potential of H3R antagonist E177 as adjuvant to antiepileptic drugs with an added advantage of preventing cognitive impairment, highlighting the H3Rs as a potential target for the therapeutic management of epilepsy with accompanied memory deficits.

## 1. Introduction

Epilepsy is a prevalent and severe chronic neurological disorder, with elevated incidence of neurological disability and mortality worldwide [1]. Epilepsy characterized by repetitive unprovoked seizures leading to devastating neurological results on the patients [2], which requires long time therapy with antiepileptic drugs (AEDs), but presently available AEDs are not capable to provide sufficient seizures control in almost one-third of the patients and also do not stop the underlying epileptogenic alterations [3]. Moreover, the consistent use of one or more AEDs will augment the epilepsy-associated comorbidities such as cognitive impairment [4]. Furthermore, it was reported that oxidative stress play a contributing part in the pathogenesis and development of seizures, which enlarges the memory and attention deficits associated with epilepsy [5,6]. 

The earlier preclinical and clinical studies have proved the strong connection between the histaminergic brain system and the seizures pathophysiology [7,8]. Previous publications have documented the role of histamine as an endogenous anticonvulsant, as histamine intracerebroventricular (i.c.v.) injections have inhibited amygdaloid kindled seizures. Moreover, intraperitoneal (i.p.) injections of compounds that increase the histamine concentration in the brain (e.g., histidine and metoprine) have prevented amygdaloid kindled seizures [7,9,10]^,^ decreased the duration of electrically-induced clonic convulsions [11], and increased the threshold of pentylenetetrazole (PTZ)-induced seizures in rodents [12]. Furthermore, brain-penetrating histamine H1 receptor (H1R) antagonists were found to induce seizures in different experimental animal models [10,13,14,15].

Interestingly, in the past decades histamine H3 receptor (H3R) antagonists/ inverse agonists obtained an enormous attention regarding their possible diverse applications in the treatment of different neuropsychiatric diseases including epilepsy, dementia, Alzheimer’s disease, autism spectrum disorder and cognitive impairment [16,17,18,19,20,21,22,23]. This owing to H3Rs unique feature by acting as autoreceptors through regulating the synthesis and release of histamine [24], in addition to acting as heteroreceptors by regulating the release of other neurotransmitters (e.g., acetylcholine, serotonin, noradrenalin and others) [23]. Moreover, other studies have indicated the protective effect of H3R antagonists/ inverse agonists in different acute seizures models including maximal electroshock (MES) and PTZ-induced seizure models [19,25,26,27]. Furthermore, the procognitive effect of H3R antagonists/ inverse agonists was confirmed lately by applying different behavioral tests including inhibitory avoidance paradigm (IAP), novel object recognition test (NOR) and Morris water maze test (MWM) [20,26,28,29,30]. In addition, we have documented the protective effect of E177, a potent non-imidazole H3R antagonist/ inverse agonist, in MES- and PTZ- induced seizure models, with E177 (10 mg/kg i.p.) showing the most promising anticonvulsant effect [31]. Also, E177 (5 mg/kg i.p.) has attenuated the memory impairment induced by dizocilpine (DIZ) in IAP and NOR tests without modulating the anxiety levels in tested rats [32]. 

The PTZ kindling model has been widely used as an experimental animal model to study the epileptogenic process, to investigate post repetitive seizures consequences on memory, cognition and oxidative stress, and to screen the effectiveness of the potential treatments for cognitive and behavioral alternations associated with epilepsy [33,34,35,36]. Interestingly, Zhang LS et al. have indicated the protective effect of thioperamide, a potent H3R antagonist, as i.c.v. chronic injections of thioperamide have significantly delayed the onset of PTZ-kindling, suppressed the seizure stages and ameliorated the spatial memory impairment associated with PTZ-kindling in rats [28]. 

In the current study and as a continuation of our work the effect of E177, the effects of the selective and potent H3R antagonist/inverse agonist on the seizure scores and memory-impairment associated with PTZ kindling were assessed. Moreover, the effects of E177 on oxidative stress markers, acetylcholine esterase (AChE) activity, levels of various brain neurotransmitters, and c-Fos protein expression were investigated in hippocampal tissues of PTZ-kindled animals. In addition, abrogative studies were carried out by co-administering CNS penetrant H3R agonist (*R*)-α-methylhistamine (RAM) to test whether brain histaminergic neurotransmissions are involved in the effects provided by the H3R antagonist E177. The H3R antagonist E177, namely [1-(6-(naphthalen-2-yloxy)hexyl)azepane] which belongs to the non-imidazole class of novel H3R antagonists, and with high antagonist affinity (*K*_i_ = 69.40 nM) and high in vitro selectivity towards H3Rs [37], was selected for the current series of experiments, as previous experiments indicated its promising anticonvulsant effect in different acute seizure models, e.g., MES- and PTZ-induced seizure models [27]. Moreover, E177 has recently shown a dose-dependent procognitive effect in DIZ-induced amnesia without modulation of anxiety-like behaviors [32], and neuroprotective effects on pilocarpine-induced status epilepticus in rodents [38].

## 2. Results

### 2.1. Effect of Chronic Administration of E177 on the Average Seizure Score of PTZ-Kindled Rats

**Figure 1:**
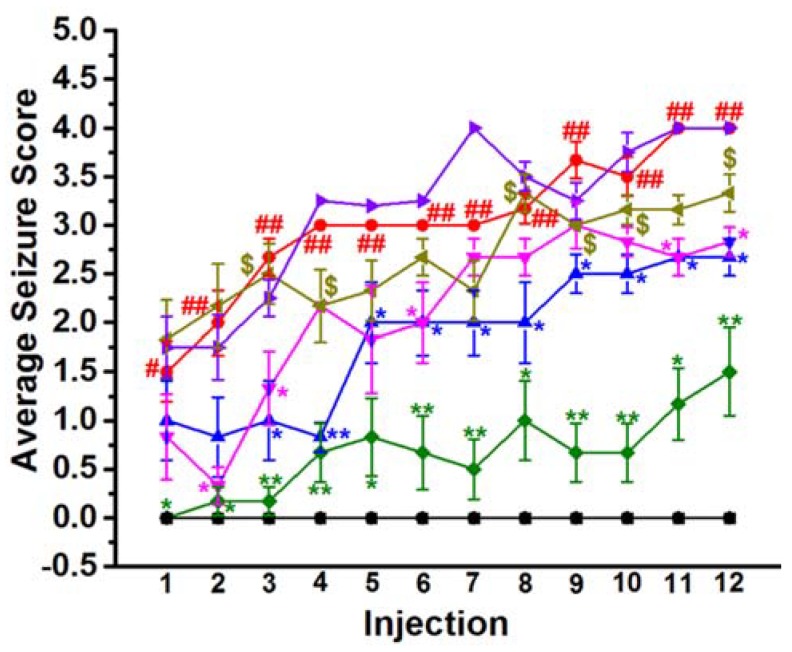
H3R antagonist E177 reduced the average seizure score of PTZ-kindled rats. Effects of PTZ (40 mg/kg), H3R antagonist E177 (5 and 10 mg/kg; i.p.) and VPA (300 mg/kg, i.p.) on average seizure score were assessed. Black lines represent SAL-treated group and the RAM-treated control group. Green line represents VPA (300 mg/kg)-treated group. Blue line represents H3R antagonist E177 (5 mg/kg)-treated group. Pink line represents H3R antagonist E177 (10 mg/kg)-treated group. Dark-yellow line represents RAM (10 mg/kg) + E177 (5 mg/kg)-treated group. Red represents PTZ (40 mg/kg)-treated group. Violet line represents RAM-treated PTZ-kindled group. Effects shown are expressed as average of seizure score for 30 min observation time for each PTZ injection. H3R antagonist E177 (5 and 10 mg/kg; i.p.) and VPA (300 mg/kg, i.p.) were administered 30–45 min prior each PTZ-injection. Effects of systemic co-injection with RAM (10 mg/kg, i.p.) on H3R antagonist E177 (5 mg/kg)-provided anticonvulsant effect was evaluated. Values are expressed as the mean ± SEM (*n* = 12). * *P* < 0.05 vs. PTZ-kindled group. ** *P* < 0.001 vs. PTZ-kindled group. ^#^ *P* < 0.05 vs. saline-treated group. ^##^ *P* < 0.001 vs. saline-treated group. ^$^ *P* < 0.05 vs. H3R antagonist E177 (5 mg)-treated PTZ-kindled rats.

Chronic animal models of seizures were established to identify the mechanisms underlying epileptogenesis and seizures production and to investigate the emotional, cognitive, and memory impairments associated with epilepsy as well as with the chronic use of several AEDs [39]. The behavioral deficits and memory impairments associated with repetitive seizures were found to be increased consensually with the severity and frequency of the convulsions [40]. The average seizure score upon each PTZ (40 mg/kg, i.p.) injection was scored and discussed in Figure 1. The results indicated that repeated administration of a subconvulsant dose of PTZ (40 mg/kg, i.p.) three times a week led to a significant increase of the average seizure score to 4 or 5 on two consecutive injections starting from the 8th injection. The statistical analysis of the obtained results revealed that the average seizure score of PTZ- treated rats increased remarkably from first injection to twelfth injection with [*F*_(1,18)_ = 47.27; *P* < 0.001] (Figure 1). Furthermore, statistical analysis indicated that the average seizure score for each PTZ injection was significantly higher when compared with SAL-treated rats, with [*F*_(1,22)_ = 19.28; *P* < 0.05], [*F*_(1,22)_ = 30; *P* < 0.001], [*F*_(1,22)_ = 160; *P* < 0.001] for 1st to 3th injection, respectively, with [*F*_(1,22)_ = 655; *P* < 0.001] for 4th to 7th injections, [*F*_(1,22)_ = 361; *P* < 0.001], [*F*_(1,22)_ = 302.5; *P* < 0.001], [*F*_(1,21)_ = 235.2; *P* < 0.001] for 8th to 10th injection, respectively, and with [*F*_(1,18)_ = 655; *P* < 0.001] for 11th and 12th injection, respectively (Figure 1). However, the results showed that chronic pretreatment with H3R antagonist E177 (5 mg/kg, i.p.) 30–45 min before each PTZ injection, caused a significant reduction of the average seizure score starting from the 3rd to 12th injection, with [*F*_(1,22)_ = 20; *P* < 0.05], [*F*_(1,22)_ = 169; *P* < 0.001], [*F*_(1,22)_ = 5; *P* < 0.05], [*F*_(1,22)_ = 7.5; *P* < 0.05], [*F*_(1,22)_ = 7.5; *P* < 0.05], [*F*_(1,22)_ = 6; *P* < 0.05], [*F*_(1,22)_ = 14.41; *P* < 0.05], [*F*_(1,21)_ = 7.68; *P* < 0.05], [*F*_(1,18)_ = 12; *P* < 0.001], and [*F*_(1,18)_ = 12; *P* < 0.05], respectively (Figure 1). When compared with PTZ-kindled rats, chronic pretreatment with H3R antagonist E177 (10 mg/kg, i.p.) caused only a significant decrease in the average seizure score following 2nd, 3rd, 6th, 11th and 12th injection, with [*F*_(1,22)_ = 15.62; *P* < 0.05], [*F*_(1,22)_ = 15.62; *P* < 0.05], [*F*_(1,22)_ = 5; *P* < 0.05], [*F*_(1,18)_ = 12; *P* < 0.05] and [*F*_(1,22)_ = 14.7; *P* < 0.05], respectively (Figure 1). Moreover, chronic pretreatment with the reference drug VPA (300 mg/kg, i.p.) 30–45 min before each PTZ injection led to a significant decrease of the average seizure score among all the injections when compared with PTZ-kindled group, with [*F*_(1,22)_ = 19.28; *P* < 0.05], [*F*_(1,22)_ = 20.86; *P* < 0.05], [*F*_(1,22)_ = 289; *P* < 0.001], [*F*_(1,22)_ = 49; *P* < 0.001], [*F*_(1,22)_ = 15.94; *P* < 0.05], [*F*_(1,22)_ = 30.62; *P* < 0.001], [*F*_(1,22)_ = 53.57; *P* < 0.001], [*F*_(1,22)_ = 20.6; *P* < 0.05], [*F*_(1,22)_ = 14.41; *P* < 0.05], [*F*_(1,21)_ = 35.56; *P* < 0.001], [*F*_(1,18)_ = 14.94; *P* < 0.05], and [*F*_(1,18)_ = 7.5; *P* < 0.05], respectively (Figure 1). Notably, no significant difference was observed for the effects of two different doses of H3R antagonist E177 (5 or 10 mg/kg) on the average seizure score of PTZ-kindled rats among all the 12th injections (*P* > 0.05) (Figure 1). Additionally, the abrogation of H3R antagonist E177(5 mg)-provided protection was evaluated by chronic co-injection with the CNS penetrant histamine H3R agonist RAM (10 mg/kg, i.p.) before each PTZ injection (Figure 1). As confirmed following statistical analysis, the results indicated that chronic co-injection with RAM led to significant increase of the average seizure score in 3rd, 4th, 8th, 9th, 10th and 12th when compared with E177(5 mg)-treated PTZ-kindled rats, with [*F*_(1,22)_ = 7.1; *P* < 0.05], [*F*_(1,22)_ = 9.41; *P* < 0.05], [*F*_(1,22)_ = 7.27; *P* < 0.05], [*F*_(1,22)_ = 5; *P* < 0.05], [*F*_(1,21)_ = 5.71; *P* < 0.05], and [*F*_(1,18)_ = 5; *P* < 0.05], respectively (Figure 1). Notably, chronic administration of RAM to PTZ-kindled or control rats failed to exhibit any significant change on the average seizure score when compared with SAL- treated PTZ-kindled rats (all *P* > 0.05). 

Moreover, the mortality rate was significantly decreased following systemic pretreatment with E177 (5 and 10 mg/kg, i.p.), with observed protective effect comparable to that observed with the reference drug VPA (Table 1). Interestingly, the E177-provided decrease in mortality rate was abrogated when rats were co-administered with the CNS penetrant H3R agonist RAM (Table 1). 

### 2.2. Effects of E177 on Mmemory Deficits in Inhibitory Avoidance Paradigm of PTZ-Kindled Rats

**Figure 2:**
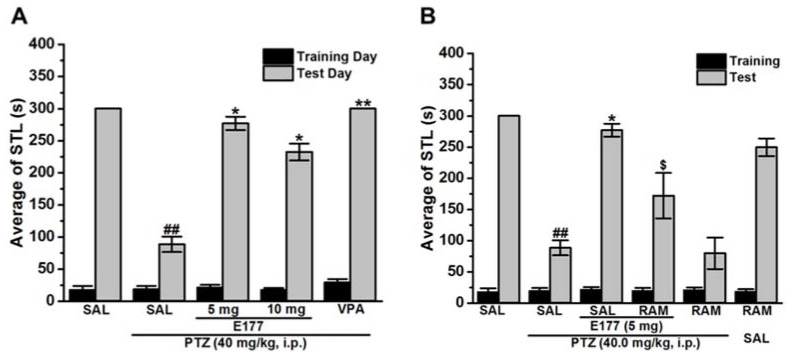
H3R antagonist E177 treatment counteracted PTZ-induced memory deficits in inhibitory avoidance paradigm. Average STL time measured on the first training day before the delivery of foot shock (black columns), and average STL time measured on the test day (gray columns). (**A**) STL time measured on the test day (gray columns). ^##^ *P* < 0.001 for average STL time compared with that of the SAL-treated group. * *P* < 0.05 for average STLs compared with the PTZ-kindled group. ** *P* < 0.001 for average STLs compared with the PTZ-kindled group. The data are expressed as the mean ± SEM (*n* = 6). (**B**) Effect of chronic co-injection of E177 (5 mg/kg) with RAM (10 mg/kg) before each PTZ injection on the average of STL time. ^$^ *P* < 0.05 for average STLs compared with the H3R antagonist E177 (5 mg)-treated rats. The data are expressed as the mean ± SEM (*n* = 6).

The retrieval memory impairment accompanied by PTZ chronic treatment was previously demonstrated in numerous previous studies in elevated plus maze (EPM) paradigm [2,33,41,42,43] and several other behavioral tests, e.g., MWM paradigm [44,45,46]. In the current study, the procognitive effect of H3R antagonist E177 (5 and 10 mg/kg, i.p.) on memory deficits in inhibitory avoidance paradigm (IAP) of PTZ-kindled male Wistar rats were evaluated (Figure 2). Statistical analysis indicated, that PTZ chronic administration significantly decreased step-through latency (STL) time when compared with SAL-treated rats, with [*F*_(1,10)_ = 22.06; *P* < 0.001]. Moreover, results of chronic pretreatment with the reference drug valproic acid (VPA, 300 mg/kg, i.p.) 30–45 min before each PTZ injection indicated a significant improvement in STL time, with [*F*_(1,10)_ = 22.06; *P* < 0.001] as compared with PTZ-kindled rats (Figure 2A). Furthermore, statistical analysis revealed that chronic systemic pretreatment with H3R antagonist E177 (5 and 10 mg/kg, i.p.) significantly counteracted the memory impairment induced in PTZ-kindled rats, with [*F*_(1,10)_ = 14.24; *P* < 0.05] and [*F*_(1,10)_ = 6.87; *P* < 0.05], respectively, and as compared with PTZ-kindled rats (Figure 2A). In addition, the results showed that no significant difference between the two doses applied (5 and 10 mg/kg) was observed by H3R antagonist E177-provided procognitive effect, with [*F*_(1,10)_ = 1.4; *P* = 0.26] (Figure 2A). Importantly, chronic co-injection with the CNS penetrant histamine H3R agonist RAM reversed the H3R antagonist E177-provided procognitive effect, with [*F*_(1,10)_ = 5.42; *P* < 0.05], and when compared with the H3R antagonist E177(5 mg)-treated rats (Figure 2B). Notably, RAM chronic administration to PTZ-kindled or control animals did not alter STL time, with [*F*_(1,10)_ = 0.02; *P* = 0.87] and [*F*_(1,10)_ = 3.6; *P* = 0.08], respectively (Figure 2B). 

### 2.3. Effect of Chronic Administration of H3R Antagonist E177 on Memory Deficits of PTZ-Kindled Rats in Elevated Plus Maze Paradigm

**Figure 3:**
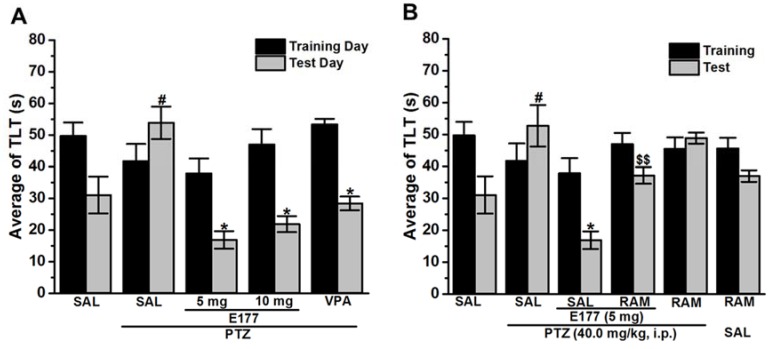
H3R antagonist E177 treatment attenuated PTZ-induced memory deficits in elevated plus maze test. Average of TLT measured on the first training day, namely 24 h after last PTZ injection (black columns), and average of TLT measured on the test day, namely 24 h after the training day (gray columns). (**A**) ^#^ *P* < 0.05 vs. saline-treated group. * *P* < 0.05 vs. PTZ-kindled group. (**B**). ^$^ *P* < 0.05 for average STLs compared with the H3R antagonist E177 (5 mg)-treated rats. ^$$^ *P* < 0.001 for average STLs compared with the H3R antagonist E177 (5 mg)-treated rats. Values are expressed as the mean + SEM (*n* = 6).

The procognitive effect of compound E177 (5 and 10 mg/kg, i.p.) on memory deficits of PTZ-kindled rats in EPMparadigm were assessed (Figure 3). Statistical analysis indicated that PTZ-kindled rats exhibited significantly increased transfer latency time (TLT) when compared with control SAL-treated rats, with [*F*_(1,10)_ = 5.16; *P* < 0.05] (Figure 3A). Moreover, results showed that chronic pretreatment with VPA (300 mg/kg, i.p.) 30–45 min before each PTZ injection significantly decreased TLT, with [*F*_(1,10)_ = 7; *P* < 0.05] when compared with PTZ-kindled rats (Figure 3A). Furthermore, statistical analysis showed that chronic pretreatment with H3R antagonist E177 (5 and 10 mg/kg, i.p.) significantly counteracted the memory impairment of PTZ-kindled animals by significantly decreasing TLT, with [*F*_(1,10)_ = 14.20; *P* < 0.05] and [*F*_(1,10)_ = 10.82; *P* < 0.05], respectively, and as compared with PTZ-kindled rats, and no significant difference was observed in the procognitive effect provided by H3R antagonist E177 (5 and 10 mg/kg), with [*F*_(1,10)_ = 1.47; *P* = 0.25] (Figure 3A). In addition, the H3R antagonist E177-provided procognitive effect was abrogated by chronic co-administration with the CNS penetrant histamine H3R agonist RAM (10 mg/kg, i.p.), with [*F*_(1,10)_ = 23.93; *P* < 0.001] when compared with H3R antagonist E177 5(mg)-treated PTZ-kindled rats (Figure 3B). Notably, RAM chronic administration to PTZ-kindled or control animals failed to alter TLT when compared with saline-treated PTZ-kindled rats or saline-treated control rats, with [*F*_(1,10)_ = 0.27; *P* = 0.61] and [*F*_(1,10)_ = 0.79; *P* = 0.39], respectively (Figure 3B). 

### 2.4. Effects of E177 Treatment on Oxidative Stress Level of PTZ-Kindled Rats

**Figure 4:**
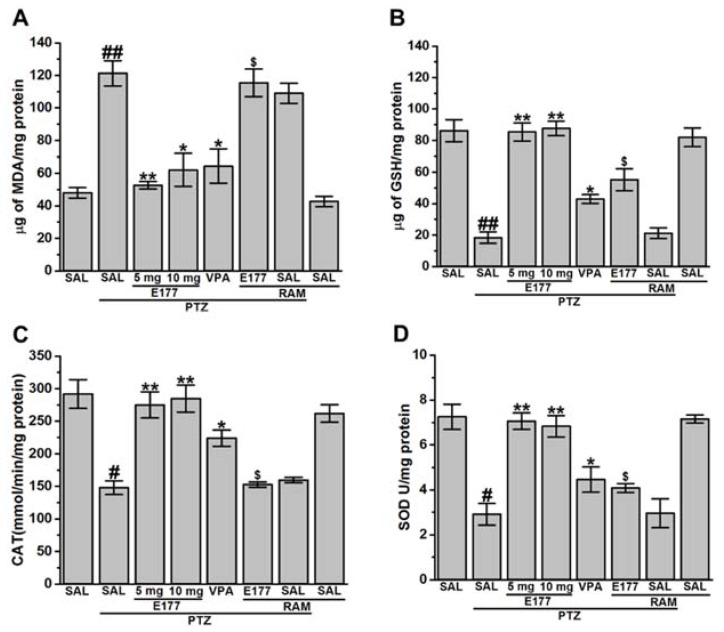
H3R antagonist E177 treatment mitigated altered oxidative stress levels associated with PTZ kindling model. Levels of glutathione (GSH), malondialdehyde (MDA), catalase (CAT), and superoxide dismutase (SOD) were assessed in rat hippocampus. PTZ-(40 mg/kg)-treated rats showed a significant decrease in GSH levels (**A**), CAT levels (**C**) and SOD levels (**D**) and a significant increase in MDA levels (**B**) when compared with saline-treated rats. E177 (5 and 10 mg/kg; i.p.) and VPA (300 mg/kg, i.p.) were administered 30–45 min prior each PTZ-injection. Effects of systemic co-injection with RAM (10 mg/kg, i.p.) on E177 (5 mg/kg)-provided modulation of oxidative stress levels were assessed. Data are expressed as the mean ± SEM (*n* = 6). ^#^ *P* < 0.05 vs. saline-treated rats. ^##^ *P* < 0.001 vs. saline-treated rats. * *P* < 0.05 vs. PTZ-kindled group. ** *P* < 0.001 vs. PTZ-kindled rats. ^$^ *P* < 0.05 vs. E177(5 mg)-treated PTZ-kindled rats.

Several antioxidant compounds were found to mitigate memory impairment in different animal models [47,48,49,50,51,52,53]. Therefore, evaluating numerous oxidative stress markers (e.g., malondialdehyde (MDA), glutathione (GSH), catalase (CAT), and superoxide dismutase (SOD)) of PTZ-kindled animals were carried out to assess the effects of the novel H3R antagonist E177. Furthermore, and amongst all the brain regions, the hippocampus has gained attention in the PTZ model as it contains several distinct neuronal circuits related to seizure genesis [54,55,56,57,58,59]. In the present study, the capability of H3R antagonist E177 to attenuate oxidative stress markers was evaluated in hippocampus tissues of PTZ-kindled rats (Figure 4A–D). Statistical analysis of the observed results indicated that MDA levels were markedly increased in PTZ-kindled rats [*F*_(1,10)_ = 63.96; *P* < 0.001], while GSH levels were significantly decreased [*F*_(1,10)_ = 16.7; *P* < 0.001] when compared with SAL-treated control rats (Figure 4A,B). Moreover, results showed a significant decrease in CAT and SOD levels in PTZ-kindled rats when compared with SAL-treated control group, with [*F*_(1,10)_ = 25.6; *P* < 0.05] and [*F*_(1,10)_ = 23.71; *P* < 0.05], respectively (Figure 4C,D). However, chronic pretreatment with H3R antagonist E177 (5 and 10 mg/kg) significantly decreased MDA levels, with [*F*_(1,10)_ = 60.47; *P* < 0.001] and [*F*_(1,10)_ = 18.35; *P* < 0.05], respectively, and increased GSH levels, with [*F*_(1,10)_ = 96.74; *P* < 0.001] and [*F*_(1,10)_ = 356.1; *P* < 0.001], respectively, and as compared with PTZ-kindled rats. In addition, chronic pretreatment with H3R antagonist E177 (5 and 10 mg/kg) significantly increased CAT levels, with [*F*_(1,10)_ = 45.06; *P* < 0.001] and [*F*_(1,10)_ = 32.91; *P* < 0.05], respectively, and SOD levels, with [*F*_(1,10)_ = 40.43; *P* < 0.001] and [*F*_(1,10)_ = 37.03; *P* < 0.001], respectively when compared with PTZ-kindled rats (Figure 4C,D). Notably, no significant difference was detected in the protective effect provided by H3R antagonist E177 (5 and 10 mg/kg), with [*F*_(1,10)_ = 0.69; *P* = 0.42], [*F*_(1,10)_ = 1.93; *P* = 0.19], [*F*_(1,10)_ = 0.01; *P* = 0.9], and [*F*_(1,10)_ = 1.6; *P* = 0.25] for MDA, GSH, CAT and SOD, respectively (Figure 4A–D). Importantly, chronic pretreatment with VPA (300 mg/kg) significantly attenuated the oxidative stress by decreasing the MDA levels [*F*_(1,10)_ = 16.18; *P* < 0.05], increasing GSH levels [*F*_(1,10)_ = 8.74; *P* < 0.05], decreasing both CAT [*F*_(1,10)_ = 16.06; *P* < 0.05] and SOD [*F*_(1,10)_ = 7.34; *P* < 0.05] levels (Figure 4A–D). Also, statistical analysis of the observed results indicated that chronic co-injection of RAM (10 mg/kg) significantly abrogated the H3R antagonist E177 (5 mg)-provided antioxidant protective effect on MDA and GSH levels, with [*F*_(1,10)_ = 7; *P* < 0.05] and [*F*_(1,10)_ = 16.06; *P* < 0.05], respectively when compared with H3R antagonist E177 (5 mg)-treated PTZ-kindled rats (Figure 4A,B). Similarly, RAM nullified the effects of E177 on CAT and SOD levels, with [*F*_(1,10)_ = 6.45; *P* < 0.05] and [*F*_(1,10)_ = 24.92; *P* < 0.05], respectively, and as compared with H3R antagonist E177 (5 mg)-treated PTZ-kindled rats (Figure 4C,D). Notably, chronic pretreatment with RAM to PTZ-kindled rats did not alter oxidative stress statues, with [*F*_(1,10)_ = 1.28; *P* = 0.28] and [*F*_(1,10)_ = 0.26; *P* = 0.62], [*F*_(1,10)_ = 0.53; *P* = 0.49], and [*F*_(1,10)_ = 0.42; p=0.54], for MDA, GSH, CAT, and SOD, respectively, and as compared with PTZ-kindled rats (Figure 4A–D). 

Moreover, chronic pretreatment of RAM to control rats also did not significantly alter oxidative stress statues, with [*F*_(1,10)_ = 1.13; *P* = 0.31] and [*F*_(1,10)_ = 0.18; *P* = 0.68], [*F*_(1,10)_ = 0.02; *P* = 0.89], and [*F*_(1,10)_ = 0.42; *P* = 0.54], for MDA, GSH, CAT, and SOD, respectively, and as compared with saline-treated control animals (Figure 4C,D).

### 2.5. Effects of H3R Antagonist E177 Treatment on Altered levels of Histamine, Acetylcholine, GABA, and Gultamate in Hippocampal Tissues of PTZ-Kindled Rats

Several preclinical studies revealed the potential memory-enhancing effect of numerous H3R antagonists [16,18,21,26,55,56,57,58,59] capable of modulating the release of brain histamine (HA) by antagonizing H3 auto- receptors, and numerous other brain neurotransmitters, e.g., acetylcholine (ACh), γ-aminobutyric acid (GABA), and glutamate (GLU) by antagonizing H3 hetero-receptors in different brain regions [60,61,62]. 

**Figure 5:**
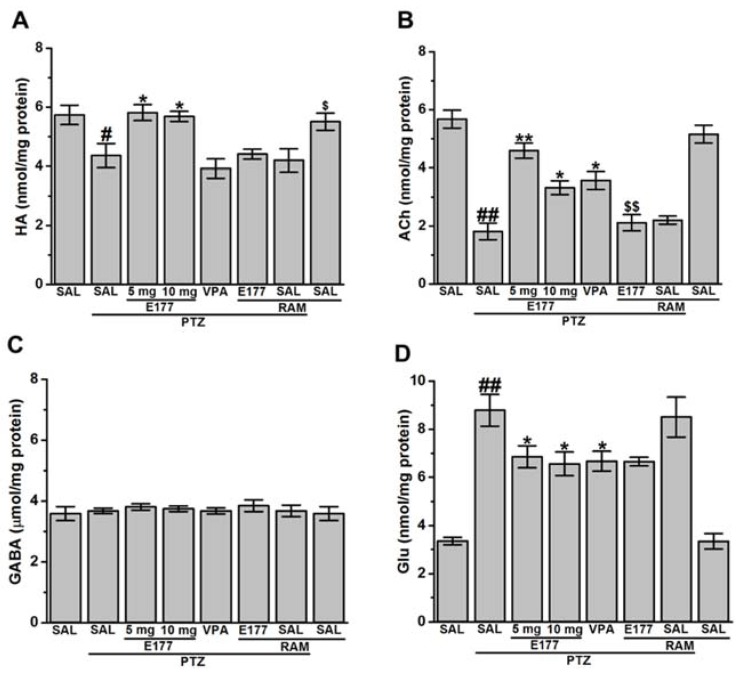
H3R antagonist E177 treatment restored altered levels of histamine, acetylcholine, GABA, and gultamate in hippocampal tissues of PTZ-kindled animals. Levels of histamine (HA), acetylcholine (ACh), γ-aminobutyric acid (GABA), and glutamate (GLU) were assessed in rat hippocampus. Effect of PTZ-(40 mg/kg), H3R antagonist E177 (5 and 10 mg/kg; i.p.) and VPA (300 mg/kg, i.p.) on HA levels (**A**), ACh levels (**B**), GABA levels (**C**) and GLU levels (**D**) were assessed. H3R antagonist E177 (5 and 10 mg/kg; i.p.) and VPA (300 mg/kg, i.p.) were administered 30–45 min prior each PTZ-injection. Effects of systemic co-injection with RAM (10 mg/kg, i.p.) on H3R antagonist E177 (5 mg/kg)-provided modulation of brain neurotransmitters levels were assessed. Data are expressed as the mean ± SEM (*n* = 6). ^#^ *P* < 0.05 vs. SAL-treated rats. ^##^ *P* < 0.001 vs. SAL-treated rats. * *P* < 0.05 vs. PTZ-kindled rats. ** *P* < 0.001 vs. (PTZ)-kindled rats. ^$^ *P* < 0.05 vs. H3R antagonist E177 (5 mg)-treated PTZ-kindled rats.

The capability of H3R antagonist E177 to modulate neurotransmitters concentration was evaluated in hippocampal tissue of PTZ- treated male Wistar rats and discussed in (Figure 5A–D). The observed results showed that chronic pretreatment with H3R antagonist E177 and VPA 30–45 min before each PTZ injection significantly modulated the average of the hippocampal levels of HA, ACh, GABA, and GLU, with [*F*_(5,30)_ = 7.56; *P* < 0.001], [*F*_(5,30)_ = 12.62; *P* < 0.001], [*F*_(5,30)_ = 218.2; *P* < 0.001], and [*F*_(5,30)_ = 15.76; *P* < 0.001], respectively. Moreover, statistical analysis of the observed results indicated that PTZ-kindled rats exhibited a significant decrease in both hippocampal levels of HA [*F*_(1,10)_ = 6.69; *P* < 0.05] and ACh [*F*_(1,10)_ = 60.87; *P* < 0.001], when compared with saline-treated PTZ-kindled rats (Figure 5A,B). Furthermore, the obtained results indicated that PTZ-kindling resulted in a significant increase of GLU [*F*_(1,10)_ = 53.16; *P* < 0.001] when compared with saline-treated control rats, whereas hippocampal GABA concentration [*F*_(1,10)_ = 0.09; *P* = 0.76] was not altered when compared with saline-treated PTZ-kindled rats (Figure 5C,D). However, chronic systemic treatment with H3R antagonist E177 (5 and 10 mg/kg) significantly increased hippocampus HA concentration, with [*F*_(1,10)_ = 6.67; *P* < 0.05] and [*F*_(1,10)_ = 6.68; *P* < 0.05], respectively, and when compared with saline-treated PTZ-kindled rats (Figure 5A). Also, chronic treatment with H3R antagonist E177 (5 and 10 mg/kg) significantly increased ACh levels in the hippocampus, with [*F*_(1,10)_ = 53.51; *P* < 0.001] and [*F*_(1,10)_ = 10.2; *P* < 0.05], respectively, and when compared with saline-treated PTZ-kindled rats (Figure 5B). Moreover, chronic treatment with H3R antagonist E177 (5 and 10 mg/kg) markedly decreased GLU brain levels, with [*F*_(1,10)_ = 11.6; *P* < 0.001] and [*F*_(1,10)_ = 5.98; *P* < 0.05], respectively, but it failed to modify hippocampal GABA concentration, with [*F*_(1,10)_ = 0.49; *P* = 0.5] and [*F*_(1,10)_ = 0.34; *P* = 0.57], respectively, and when compared with saline-treated PTZ-kindled rats (Figure 5C,D). Notably, no significance difference was observed between the two doses (5 and 10 mg/kg) of H3R antagonist E177 in the modulating effect of HA, ACh and GLU levels with [*F*_(1,10)_ = 0.12; *P* = 0.74], [*F*_(1,10)_ = 0.66; *P* = 0.44], and [*F*_(1,10)_ = 0.31; *P* = 0.59], respectively. Importantly, chronic treatment with the reference drug VPA (300 mg/kg) failed to change hippocampal HA and GABA levels, with [*F*_(1,10)_ = 0.66; *P* = 0.44] and [*F*_(1,10)_ = 0.66; *P* = 0.44], respectively (Figure 5A,C). However VPA chronic treatment enhanced ACh brain levels and decreased GLU levels, with [*F*_(1,10)_ = 10.15; *P* < 0.05] and [*F*_(1,10)_ = 6.07; *P* < 0.05], respectively (Figure 5B,D). Additionally, statistical analysis of the observed results indicated that chronic co-injection of RAM (10 mg/kg) abrogated the enhancement effect provided with H3R antagonist E177 (5 mg/kg) on hippocampal brain levels of HA and ACh, with [*F*_(1,10)_ = 21.18; *P* < 0.05] and [*F*_(1,10)_ = 35.53; *P* < 0.001], respectively; however, it failed to reverse the effects provided on GLU brain levels, with [*F*_(1,10)_ = 2.46; *P* = 0.14], when compared with H3R antagonist E177 (5 mg)-treated PTZ-kindled rats (Figure 5A–D). Notably, chronic pretreatment with RAM neither altered HA levels of PTZ-kindled as well as saline-treated control rats, with [*F*_(1,10)_ = 0.06; *P* = 0.80] and [*F*_(1,10)_ = 0.6; *P* = 0.47], respectively, nor ACh levels, with [*F*_(1,10)_ = 1.04; *P* = 0.34] and [*F*_(1,10)_ = 0.84; *P* = 0.39], respectively, and as compared with PTZ-kindled or control rats (Figure 5A,B). 

### 2.6. Effects of H3R Antagonist E177 Treatment on Elevated Acetylcholine Esterase Activity Levels in Hippocampus of PTZ-Kindled Rats

**Figure 6:**
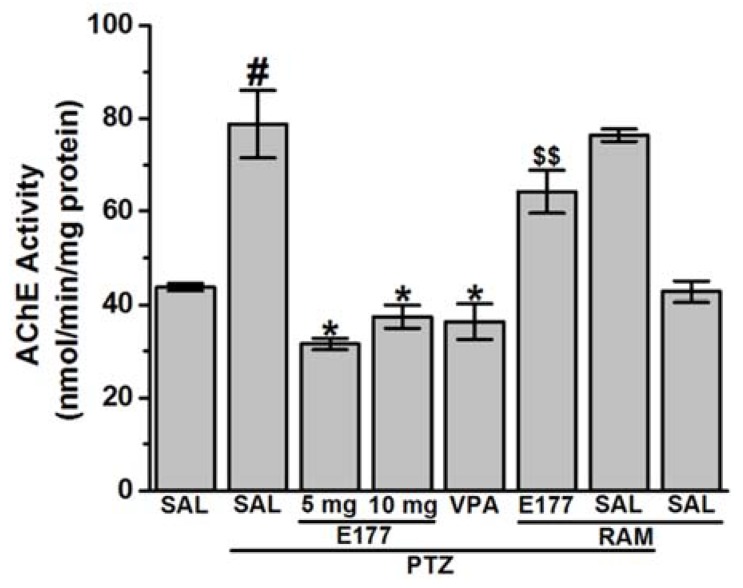
H3R antagonist E177 treatment restored elevated acetylcholine esterase activity levels in hippocampus of PTZ-kindled rats. Levels of AChE were assessed in rat hippocampus. E177 (5 and 10 mg/kg; i.p.) and VPA (300 mg/kg, i.p.) were administered 30–45 min prior each PTZ-injection. Effects of systemic co-injection with RAM (10 mg/kg, i.p.) on E177 (5 mg/kg)-provided modulation on AChE levels were assessed. Data are expressed as the mean ± SEM (*n* = 6). ^#^ *P* < 0.05 vs. saline-treated rats. * *P* < 0.05 vs. PTZ-kindled rats. ^$$^ *P* < 0.001 vs. E177(5 mg)-treated PTZ-kindled rats.

Acetylcholinesterase enzyme (AChE) is an essential enzyme that hydrolysis the ACh and terminates the cholinergic transmission. Therefore, AChE is considered as an important therapeutic target, and numerous reversible inhibitors of AChE are being clinically used as a memory enhancer in epileptic patients and other neurodegenerative diseases [63,64]. In our study, the statistical analysis of the observed results indicated that PTZ-kindling led to a significant increase of hippocampal levels of AChE [*F*_(1,10)_ = 12.82; *P* < 0.05] when compared to control rats (Figure 6). Chronic pretreatment with H3R antagonist E177 (5 and 10 mg/kg, i.p.) significantly decreased the elevated hippocampal AChE levels, with [*F*_(1,10)_ = 22.67; *P* < 0.05] and [*F*_(1,10)_ = 15.39; *P* < 0.05], respectively, and when compared with PTZ-kindled rats, and without significant difference detected in the protective effect provided by H3R antagonist E177 (5 and 10 mg/kg) [*F*_(1,10)_ = 1.83; *P* = 0.21] (Figure 6). Moreover, chronic pretreatment with the reference drug VPA (300 mg/kg, i.p.) resulted in a significant decrease of hippocampal AChE levels [*F*_(1,10)_ = 14.0; *P* < 0.05] (Figure 6). Additionally, statistical analysis of the observed results indicated that chronic co-injection of RAM (10 mg/kg) significantly counteracted the H3R antagonist E177 (5 mg)- provided protective effect on AChE levels [*F*_(1,10)_ = 47.73; *P* < 0.001], and when compared with H3R antagonist E177 (5 mg)-treated PTZ-kindled rats (Figure 6). Notably, chronic pretreatment with RAM to PTZ-kindled rats did not alter AChE levels, with [*F*_(1,10)_ = 1.8; *P* = 0.20] and [*F*_(1,10)_ = 0.3; *P* = 0.6], respectively, and as compared to PTZ-kindled or saline-treated control rats (Figure 6). 

### 2.7. Effects of H3R Antagonist E177 Treatment on Levels of c-Fos Protein Expression in Hippocampus of PTZ-Kindled Rats

**Figure 7:**
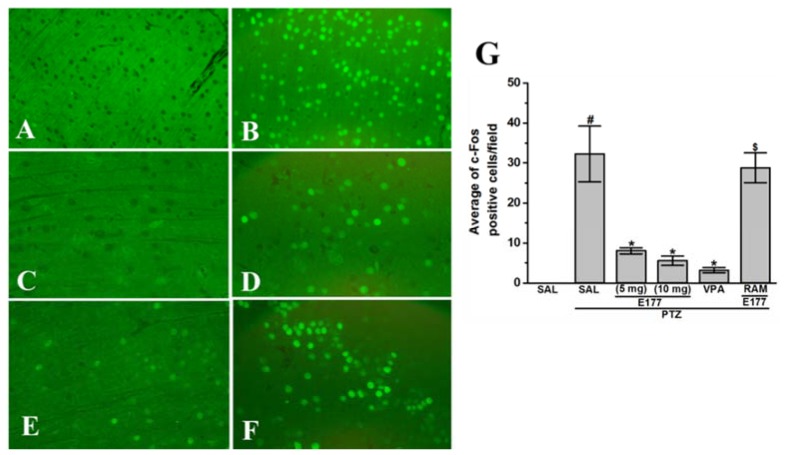
H3R antagonist E177 treatment mitigated altered levels of c-Fos protein expression in hippocampus of PTZ-kindled rats. Profound expression of c-Fos protein in the hippocampus of PTZ-kindled rats (**B**) when compared to saline-treated control rats (**A**). Quantitative analysis (**G**) of c-Fos positive cells revealed that reference drug VPA (300 mg/kg, (**C**)) and H3R antagonist E177 (5 and 10 mg/kg, (**D**,**E**)) significantly mitigated the increased levels of c-Fos in PTZ-kindled rats. Chronic systemic co-injection of RAM (10 mg/kg, i.p) counteracted the E177 (5 mg)-provided amelioration of c-Fos-expression of PTZ-kindled rats (**F**). ^#^ *P* < 0.05 for average c-Fos positive cells compared with saline-treated control group. * *P* < 0.05 for average c-Fos positive cells compared with PTZ-kindled rats. ^$^ *P* < 0.05 for average c-Fos positive cells vs. H3R antagonist E177 (5 mg)-treated PTZ-kindled rats. Data are expressed as mean ± SEM (*n* = 6).

Comorbidities accompanied by epilepsy are not only related to a single complication, as there is a collective matrix of oxidative stress, neurochemical changes, and histological alterations in the brain which cooperatively provoke comorbidities [65,66,67]. The protein c-Fos is a transcription factor that belongs to the immediate early genes (IEGs) family that is rapidly activated post numerous cellular incentives. The observed results showed that chronic systemic administration of PTZ (40 mg/kg, i.p.) significantly increased the number of c-Fos positive hippocampal cells in rats, with [*F*_(1,10)_ = 17.58; *P* < 0.05] when compared with control rats (Figure 7A,B). 

However, chronic pretreatment with the reference drug VPA (300 mg/kg) and the H3R antagonist E177 (5 and 10 mg/kg) significantly reduced the number of c-Fos positive cells, with [*F*_(1,10)_ = 7.97; *P* < 0.05], [*F*_(1,10)_ = 9.95; *P* < 0.05], and [*F*_(1,10)_ = 11.52; *P* < 0.05], respectively, when compared with saline-treated PTZ-kindled rats, and without significant difference detected in the protective effect provided by H3R antagonist E177 (5 and 10 mg/kg) [*F*_(1,10)_ = 2.55; *P* = 0.15] (Figure 7C–E). Additionally, the statistical analysis of the observed results indicated that chronic co-injection of RAM (10 mg/kg) 20 min prior to each PTZ injection abrogated the enhancement effect provided with H3R antagonist E177 (5 mg/kg) on hippocampus brain c-Fos expression [*F*_(1,10)_ = 19.92; *P* < 0.05] (Figure 7F).

## 3. Discussion

In the current study, the results showed that rats chronically pretreated with PTZ (40 mg/kg, i.p.) three times a week for 12 injections showed increased average of seizure severity score when compared to control animals, and gradually exhibited generalized tonic-clonic convulsions starting from 9th injection which eventually resulted in kindled rats (Figure 1). These results are in agreement with previous studies in which PTZ was applied for kindling of rats [68,69] and in numerous other rodent species [39,68,70,71,72]. However, chronic systemic pretreatment with the H3R antagonist E177 (5 and 10 mg/kg, i.p.) significantly prevented the kindling procedure, reduced the average seizure score, and decreased the mortality of PTZ-kindled rats, with no significant difference observed for the average seizure score following systemic administration of both doses of test compound E177 (Figure 1). Furthermore, the protective effect of H3R antagonist E177 (5 mg/kg) was reversed when rats were co-injected with the CNS-penetrant H3R agonist RAM, indicating that the H3R antagonist E177-provided protective effect was mediated through modulation of histaminergic neurotransmission by H3Rs (Figure 1). These observations are in line with previous preclinical studies in which histidine, clobenpropit, and carnosine (a precursor of histidine) significantly and dose-dependently prolonged the latency to myoclonic jerks and generalized clonic seizures in PTZ-kindled rodents, and the protective effect provided was partially abrogated with co-injection with RAM [73,74,75,76]. 

In our study, the PTZ-kindled rats showed associated memory impairments in both IAP and EPM assessments, as PTZ chronic treatment resulted in a significant decrease of STL time in IAP and a significant increase in TLT time in EPM (Figure 2 and Figure 3). Interestingly, chronic systemic pretreatment with H3R antagonist E177 (5 and 10 mg/kg) significantly mitigated the memory impairment associated with chronic administration of PTZ (Figure 2 and Figure 3). The simultaneous anticonvulsant and procognitive effects detected for the novel H3R antagonist E177 were in harmony with earlier studies in which the old-generation H3R antagonist thioperamide displayed concomitant antiepileptic and memory-enhancing effect in rats [28]. Also, thioperamide was found to attenuate memory deficits assessed in the IAP paradigm for PTZ-kindled mice [77]. In addition, the procognitive effect of H3R antagonist E177 (5 mg/kg) in IAP and EPM paradigms was nullified when rats were co-injected with RAM, indicating that modulated histaminergic neurotransmission is powerfully involved in the E177-provided procognitive effect (Figure 2 and Figure 3). Interestingly, systemic administration of H3R agonist alone failed to modulate the memory function of tested PTZ-kindled animals, excluding any confounding factors for the agents used to abrogate the E177-provided memory enhancing effects (Figure 2 and Figure 3). Accordingly, H3R antagonist E177 protected animals against seizure and, therefore, mitigated the memory impairment induced by repetitive injections of PTZ by antagonizing the H3 auto-receptors and the resulted increase in brain HA release. The latter assumption was reinforced by earlier preclinical experiments in which injection of histidine increased brain HA level and exhibited a memory-enhancing effect in PTZ-kindled rodents [78,79,80]. Importantly, the reference drug VPA, also, attenuated the memory deficits associated with PTZ-kindling of test animals (Figure 2 and Figure 3). The memory-enhancement effect of VPA in PTZ-kindled animals was previously reported in a preclinical study, whereas another AED, namely phenytoin, failed in the same study to provide any memory-enhancing effect [43]. Oxidative stress is a foundation for several neurological and neurodegenerative disorders, and it has been involved in the pathogenesis of epilepsy [81]. 

Also, oxidative stress was found to accompany neuronal hyperexcitation produced by different CNS diseases (e.g., epilepsy) [82]. Our observations showed increased level of MDA and decreased levels of GSH, CAT and SOD in the hippocampus of PTZ-kindled rats (Figure 4A–D). These results were in line with previous preclinical [34,83,84] as well as clinical observations in which an increased level of hippocampal oxidative stress was noted [85,86,87]. Our results indicated that chronic systemic pretreatment with the H3R antagonist E177 (5 and 10 mg/kg) significantly mitigated oxidative stress of PTZ-kindled rats (Figure 4A–D), and the protective effect observed for E177 (5 mg/kg) on MDA, CAT, and SOD was reversed when rats were co-injected with the H3R agonist RAM, demonstrating the involvement of the brain HA (released through antagonizing of H3Rs) in facilitating the neuroprotective action of H3R antagonist E177 in PTZ-kindled rats (Figure 4A,C,D). 

Our results indicated that PTZ-kindled rats showed a significant decrease of hippocampal HA level (Figure 5A). The observed results were consistent with previous studies in which hippocampal HA levels were decreased in fully kindled rats [78,79], demonstrating the anticonvulsant effect of the endogenous HA, a finding that has previously been confirmed in several preclinical studies [8,88,89]. Moreover, the vital role of brain HA in the pathogenesis of convulsions [90,91] and learning and memory was previously recognized [92]. Interestingly, chronic systemic pretreatment with H3R antagonist E177 (5 and 10 mg/kg) restored the depleted HA levels in hippocampus (Figure 5A), comprehending the proposed mechanism of action of the centrally acting test compound E177, as antagonism of H3 auto-receptors is believed to enhance brain HA biosynthesis and release [22,24]. Notably, previous *in-vitro* studies indicated that several H3R antagonists, e.g., thioperamide, ciproxifan, clobenpropit, A-304121, and A-317920 increased HA release in cortical brain slices [93]. Moreover, former *in-vivo* preclinical studies in different rodents indicated the influence of several H3R antagonists, e.g., thioperamide, ciproxifan, and GT-2016, in increasing brain HA release [94,95,96,97,98]. In addition, the provided enhancement effect of hippocampal HA release by our H3R antagonist E177 (5 mg/kg) was partially abrogated when rats were co-injected with RAM, confirming that the protective effect provided by H3R antagonist E177 was facilitated through the histaminergic system (Figure 5A). Similarly, PTZ-kindled rats displayed significant decrease in hippocampal levels of ACh (Figure 5B), a neurotransmitter of vital role in controlling memory, attention, and learning [99]. Our results were in line with previous studies in which PTZ decreased the concentration of hippocampal ACh in rats [77,100,101,102] and zebrafish [103,104]. However, chronic pretreatment with H3R antagonist E177 (5 and 10 mg/kg) significantly restored the depleted hippocampal ACh concentration, indicating the modulating effect of H3R antagonists E177 on cholinergic transmission function (Figure 5B). The modulating effect of H3R antagonists (e.g., thioperamide, JNJ-10181457, ABT-239, GSK189254, and ciproxifan) on brain ACh levels of several rodents was previously revealed [92,105,106,107,108,109,110]. The E177-provided modulating effect on the hippocampal ACh levels was entirely abrogated following systemic co-injection with the H3R agonist RAM (Figure 5B). Moreover, the hippocampal GLU/GABA ratio was elevated in PTZ-kindled rats (Figure 5C,D). It was suggested that kindling-induced epileptogenesis is a consequence of an imbalance among inhibitory and excitatory activities representing an enhanced glutaminergic transmission or decline in GABAergic transmission in both humans and animals [111,112,113]. GABA is an essential inhibitory neurotransmitter [9], and it was noted earlier that the proconvulsant effects of PTZ are primarily owing to its inhibitory effects on GABAergic neurotransmission [114,115]. Our current observations revealed that hippocampal GABA levels of PTZ-kindled rats remained unaffected, and this finding is, also, in agreement with previous studies which showed that chronic PTZ administration failed to modulate GABA levels in hippocampus and cortex of kindled animals [116,117]. In addition, and in a previous study, GABA levels were not found to be altered in several brain regions post PTZ-kindling, including the hippocampus [118]. Also, most of clinically accessible AEDs work through GABAergic mechanisms, therefore, effectively controlling seizures, but these AEDs were unsuccessful in attenuating the accompanied memory deficits in epileptic patients [119]. Consequently, GABAergic transmission is not the most appropriate contributor to memory deterioration associated with PTZ-kindling model. GLU is the main excitatory neurotransmitter and had been widely involved in synaptic plasticity underlying memory function [120]. However, high abnormal levels of GLU were associated with epilepsy or Alzheimer’s diseases, as an increased level of brain GLU is considered neurotoxic. Consequently, high GLU brain levels may be a contributory cause for memory deficits, e.g., in schizophrenia patients, accompanied by the uncontrolled seizures [121,122,123]. Several former preclinical experiments indicated high GLU levels in different brain regions of PTZ-kindled rodents [35,43,124,125,126], and these results are in harmony with our observations (Figure 5D). Moreover, the results observed indicated a swing in the equilibrium among excitatory and inhibitory actions toward excitation in PTZ-kindled rats, resulting in a high neuronal activity and consequently in a decreased seizure threshold and memory deficits. The neurotransmitters alternation noted in our study in PTZ-kindled rats (decline in hippocampal HA and ACh levels and increase in GLU levels) when compared with control rats, might be responsible for the observed reduction in seizure threshold of the kindled rats and the observed behavioral changes including memory impairments. Interestingly, chronic systemic pretreatment with the H3R antagonist E177 (5 and 10 mg/kg) led to a significant decrease of the abnormal high levels of hippocampal GLU in PTZ-kindled rats (Figure 6). These observations are in line with earlier results in which the H3R antagonist ciproxifan decreased the hippocampal GLU release in rats [127]. Notably, systemic co-injection with RAM failed to reverse the modulating effect of H3R antagonist E177 (5 mg/kg) on hippocampal GLU levels, suggesting that the hippocampal GLU modulating effect provided by H3R antagonist E177 was not only mediated through histaminergic transmission, but other possible mechanisms may have contributed (Figure 6). It can be suggested from earlier findings that H3R antagonists may modify neurotransmitters release differently under normal or stimulated conditions [128], which explains the inhibitory effect of H3R antagonist E177 on the PTZ-induced high GLU release through H3 hetero-receptors. Accordingly, reducing the abnormal hippocampal GLU levels was at least partially involved in the anticonvulsant and memory-enhancing effect of H3R antagonist E177 in PTZ-kindled animals. Our observations showed that chronic systemic pretreatment with VPA (300 mg/kg) 30–45 min before each PTZ injection reduced the hippocampus elevated levels of AChE (Figure 5). These results agreed with previous preclinical observations in which VPA treatment reduced the aberrant AChE levels in different brain regions of PTZ-kindled rodents, and the modulated effect was accompanied by mitigation of the associated memory impairment [3,43]. Importantly, our results indicated that H3R antagonist E177 (5 and 10 mg/kg) restored disturbed levels of AChE in the hippocampus, with no significant difference noted in the modulating effect between the two doses used (Figure 5). The H3R antagonist E177 was not confirmed in its in vitro profile of having AChE inhibitory effects [38,39]. However, the modulatory effect of H3R antagonist E177 on AChE levels may be due to its provided anticonvulsant effect which resulted in an inhibition of the AChE overexpression and/or preventing of the notable alternation of cholinergic transmission. The latter hypothesis is supported by previous studies in which normalizing the abnormal brain levels of AChE of kindled rodents by numerous compounds was associated with memory-enhancing effect [34,129,130,131]. Interestingly, the modulatory provided effect by H3R antagonist E177 (5 mg/kg) was reversed with RAM co-injection, suggesting that the modulatory effect of H3R antagonist E177 on hippocampal AChE levels is related to the anticonvulsant provided effect, and facilitated through the released HA. IEGs are able to detect stimulated neurons of seizure activity and other excitatory provocations. Moreover, IEGs is believed to be implicated in the neuronal excitation and to perform an essential role in the development of kindling process [132,133]. The basal expression of brain c-Fos is low; however, c-Fos had been noted to be activated rapidly in reaction to many incentives such as seizures induction [134], making c-Fos a valuable indicator of neuronal activity [132,135]. It was documented that c-Fos activation was highly expressed in the hippocampus upon the development of tonic-clonic convulsions (seizure score 4-5) in PTZ-kindled rodents implying that only high neuronal induction was needed to boost c-Fos expression in the hippocampus [136,137,138]. Consequently, analysis of the expression of c-Fos in the hippocampus following PTZ-kindling is used to examine hippocampal neuronal activity. Our results indicated that c-Fos protein expression was significantly increased in the hippocampus of PTZ-kindled rats, indicating hippocampal neuronal excitation (Figure 7B). Accordingly, the PTZ-induced hippocampal excitability can be correlated to the associated memory deficits of kindled rats. The observed results indicated that chronic treatment with VPA (300 mg/kg) significantly reduced hippocampal c-Fos expression of PTZ-kindled rats (Figure 7C), which supports previous observations in which VPA attenuated flurothyl- induced hippocampal c-Fos expression in male Wistar rats [139], indicating the neuroprotective effect of VPA. Similarly, chronic systemic pretreatment with H3R antagonist E177 (5 and 10 mg/kg) mitigated hippocampus c-Fos expression of PTZ-kindled rats (Figure 7D,E). These observed findings were supported by a previous study in which H3R antagonist ciproxifan alleviated the stress-evoked increase of cortex c-Fos expression in mice and attenuated the associated memory deficits [140,141]. Moreover, and in a previous study, the H3R antagonist thioperamide mitigated haloperidol-evoked striatum c-Fos expression in rats [141]. Furthermore, the effect of H3R antagonist E177 on c-Fos expression was reversed by co-injection with H3R agonist RAM, signifying the involvement of the brain histaminergic neurotransmission system in the provided neuroprotective effect (Figure 7F). 

## 4. Materials and Methods

### 4.1. Animals

Experimental inbred male Wistar rats (Central Animal Facility of UAE University) weighing between 140-160 g was used in this study. All animals were maintained in an air-conditioned animal facility room with controlled temperature (24 °C ± 2 °C) and in humidity (55% ± 15%) under a-12 light/dark cycle; all animals had free access to food and water. All experiments of this study were done between 9:00 am to 13:00 pm and all procedures were performed according to the guidelines of the European Communities Council Directive of November 24, 1986 (86/609/EEC) and was approved for the epilepsy study by the College of Medicine and Health Sciences/United Arab Emirates University (Institutional Animal Ethics Committee, approval number: ERA-2017-5676). All efforts were made to minimize animal suffering and to reduce the number of animals used. In addition, all behavioral studies were carried out by the same experimenter and in a blind manner.

### 4.2. Drugs

The H3R antagonist E177 was synthesized by the Department of Technology and Biotechnology of Drugs (Kraków, Poland) as previously described [36]. Valproic acid (VPA, 300 mg/kg, i.p), pentylenetetrazole (PTZ, 40 mg/kg, i.p.), and (*R*)-α-methylhistamine (RAM, 10 mg/kg, i.p.) were purchased from Sigma-Aldrich (St. Louis, MI, USA). All compounds were i.p. injected after dissolving with isotonic saline at the volume 1 mL/kg. VPA (300 mg/kg) was used as positive control drug because of its high antiepileptic effect and less harmful effect on memory impairment [142]. VPA anticonvulsant effect observed in our study was documented earlier in numerous publications in PTZ-kindling model [3,131,143]. 

### 4.3. In Vivo Tests 

#### 4.3.1. Kindling Procedure

Twelve animals were injected a subconvulsive dose of PTZ (40 mg/kg i.p.) three times a week for 12 injections. The PTZ injections were stopped when animals are fully kindled upon the development of seizure score 4 or 5 in three consecutive injections. All animals were observed for 30 min after each PTZ injection and the average seizure score for each rat was recorded according to the following Racine scale: 0 = no response, 1 = hyperactivity, restlessness, and eye or facial twitches, 2 = convulsive waves across the body, 3 = myoclonic jerks or rearing, 4 = turn over onto one side position, and 5 = turn over onto back position, generalized tonic-clonic seizures, or die during the experiment period. The kindling and survival rates for each group were calculated as previously described and are highlighted in Table 1 [3,131,143]. Following PTZ-kindling, eight groups of six animals were taken for IAP behavioral test, then were sacrificed and brains and tissues were prepared for the assessment of oxidative stress, neurotransmitters, and AChE activity as shown below. Another eight groups of six animals were taken for EPM behavioral test, then sacrificed and brains and tissues were prepared for the evaluation of c-Fos protein expression following the protocols explained below. 

#### 4.3.2. Experimental Design

Animals were randomly divided into eight groups of six animals. The eight groups received i.p. injections three times for the duration of four weeks, and injections were as follows; group 1 served as control group and received saline injection (i.p.), group 2 received PTZ injection (40 mg/kg i.p.), group 3 received VPA (300 mg/kg i.p.) 30–45 min before PTZ injection, and groups 4 and 5 received the test compound E177 (5 and 10 mg/kg i.p., respectively) 30–45 min before PTZ injection, group 6 received the test compound E177 (5 mg/kg i.p.) and RAM (10 mg/kg, i.p.) 30–45 min before PTZ injection, group 7 received the RAM (10 mg/kg, i.p.) 30–45 min before PTZ injection, and group 8 (control group) received RAM (10 mg/kg, i.p.) 30–45 min before saline injection. Following the IAP behavioral test, the animals were sacrificed and the whole brain was dissected for estimation of markers of oxidative stress (glutathione (GSH), malondialdehyde (MDA), catalase (CAT), and superoxide dismutase (SOD)), acetylcholine esterase (AChE) activity, and levels of several brain neurotransmitters (acetylcholine, histamine, GABA, and glutamate). Another eight groups of 6 animals were taken and received the same aforementioned treatments. Following the EPM behavioral test, the animals were sacrificed and the whole brain was dissected for estimation of c-Fos protein expression.

#### 4.3.3. Behavioral Tests

The memory impairment was assessed applying inhibitory avoidance paradigm (IAP) and elevated plus maze (EPM) 24 h after the last PTZ injection. Different animals were used for each behavioral test. 

##### Single-Trial Inhibitory Avoidance Paradigm

Memory deficit was assessed by step through passive avoidance apparatus (Step-through Cage, 7550; Ugo Basile, Comerio, Italy), as described previously [20,26,32,59,144]. The experiment consists of two trials (training and testing), in the training day each rat was placed in the white compartment and after 30 s habituation time the door was raised automatically and once the rat with all his four paws entered the dark compartment the door was closed and a foot shock of 0.4 mA (20 Hz, 8.3 ms) was delivered to the grid floor for 3 s, and STL time was measured. Immediately after the foot shock rats were returned to its home cage. Rats that had step through latency more than 60 s were excluded from the experiment. 24 h later, in the test day STL time was measured in the same way with no foot shock, however the cut off time was increased to 300 s. The reduction of STL time was used as an indicator of impaired memory [20,26,32,59,144].

##### Elevated Plus Maze

During the acquisition trial in the first day each rat was placed at the distal ending of one of the open arms facing away from central platform. TLT, the time required for the rat to enter any of the closed arms with its four paws, was measured with a cut off time at 60 s. After the animal entered the closed arm by 10 s the rat was returned to its home cage. Memory retention was examined 24 h later, applying the same protocol of the first day. Prolonged retention TLT was considered as an indicator of impaired memory [21,29,57].

### 4.4. Biochemical Estimations

The day after (24 h) the IAP behavioral test the rats were anesthetized with pentobarbital (40 mg/kg body weight), a cardiac perfusion was carried out using 0.01 M phosphate-buffered saline (PBS) at pH 7.4 in order to flush the blood out. Brains were quickly removed and placed on an ice-plate where each brain was cut in two hemispheres and immediately frozen in liquid nitrogen for further use.

#### 4.4.1. Tissue Preparation

The hemisphere was homogenized in KCl buffer (Tris–HCl, 10 mM NaCl, 140 mM KCl, 300 mM EDTA, 1 mM Triton-X-100 0.5 %) at pH 8.0 supplemented with protease and phosphatase inhibitor. The homogenate was centrifuged at 10,000× *g* for 30 min at 4 °C. The supernatant was used according to previously described experimental protocols in from our laboratories for estimation of oxidative stress markers, namely malondialdehyde (MDA), glutathione (GSH), catalase (CAT) and superoxide dismutase (SOD), and of acetylcholinesterase (AChE) activity [21,29,145,146]. 

#### 4.4.2. Measurement of MDA

The levels of lipid peroxidation product, MDA, were measured using MDA detection kit (North West Life Science, Vancouver, WA, USA). Incubation of the samples and standards (150 μL) with acid reagent and thiobarbituric acid (150 μL) for 60 min at 60 °C, after that all of the samples and standards were centrifuged for 2 min at 10,000× *g*. Then all the reactions solutions were transferred to 96 well plate reader to record the absorbance at 532 nm. The results are expressed as μM MDA/mg protein [21,29,145,146].

#### 4.4.3. Measurement of GSH

The levels of GSH was measured using a commercially available GSH kit. First, all the samples were deproteinized with 5% 5-sulfosalicylic acid solution, after that the samples were centrifuged at 10,000× *g* for 10 min in order to eliminate any precipitated protein. Then the supernatant was used in this GSH measurement. Incubation of all the samples and standards (10 μL) in 96 well plate with (150 μL) working reagent (assay buffer +5, 5′-dithiobis (2-nitrobenzoic acid) + GSH reductase) for 5 min, then it was diluted with NADPH solution (50 μL). The absorbance of the samples and standards were recorded at 412 nm with the kinetics for 5 minutes by using the micro plate reader. The results are expressed as μM GSH/mg protein [21,29,145,146]. 

#### 4.4.4. Measurement of CAT

The activity of the antioxidant enzyme, catalase (CAT) was estimated using commercially available assay kit (Cayman Chemical Company, Ann Arbor, MI, USA). Briefly, CAT was estimated by adding (20 µL) samples or standards of different concentrations with (100 µL) assay buffer and (30 µL) methanol in a 96-well plate. The reaction was initiated by adding H_2_O_2_ (20 µL) to each well and the 96-well plate was covered and incubated on a shaker for 20 min at room temperature (RT). Potassium hydroxide (30 µL) was used to terminate the reaction and subsequently (30 µL) CAT purpald were added, and then the 96-well plate was covered and incubated on a shaker for 10 min at RT. After that, (10 µL) CAT potassium periodate were added to each well. The plate was covered and incubated for 5 min on a shaker at RT. Finally, absorbance was read at 540 nm using a micro plate reader [21,29,145,146].

#### 4.4.5. Measurement of SOD

The activity of the antioxidant enzyme, superoxide dismutase (SOD) was estimated using commercially available assay kit (Cayman Chemical Company). Briefly, SOD was measured by adding (10 µL) diluted samples or standards of different concentration with (200 µL) radical detector in a 96-well plate. The reaction was initiated by adding (20 µL) xanthine oxidase to each well, then the 96-well plate was covered and incubated on shaker at RT for 30 min. Finally, absorbance was read at 450 nm using a micro plate reader [21,29,145,146].

### 4.5. Estimation of Brain Neurotransmitters

Tissue preparation was carried out following the same protocol under Section 4.4.1.

#### 4.5.1. Estimation HA level in Hippocampus of PTZ-Kindled Rats

The hippocampus HA concentration was estimated using a colorimetric assay kit (Abcam, Cambridge, UK). The protocol of the assay was followed, as described in the kit datasheet. All samples and standards with different concentrations were added to the wells in 96-well plate (10 μL), and each well was brought to (50 μL) by HA assay buffer. The reaction started by adding (50 μL) of the working reagent (HA assay buffer, HA probe, and HA enzyme mix), and the plate was incubated for 30 min at 37 °C. After that, the absorbance was recorded at 450 nm. The assay used a standard curve of HA to calculate the amount of HA in the samples. HA concentration was expressed as nmol/mg.

#### 4.5.2. Estimation ACh Level in Hippocampus of PTZ-Kindled Rats

ACh concentration was estimated using a colorimetric assay kit (Abcam). The protocol of the assay was followed, as described in the kit datasheet. All samples in duplicate and standards with different concentrations were added to the wells in 96-well plate (10 μL), and each well was brought to (50 μL) by choline assay buffer. The reaction started by adding (50 μL) of the working reagent (choline assay buffer, choline probe, and choline enzyme mix) in the first group of the samples to measure the free choline, while in the other group same working reagent was added with addition of acetylcholinesterase to measure the total choline, afterword the plate was incubated for 30 min at RT. Finally, the absorbance was recorded at 570 nm. The assay used a standard curve of choline to calculate the amount of choline in the samples. ACH concentration was calculated using the equation: total choline- free choline. ACH concentration was expressed as nmol/mg.

#### 4.5.3. Estimation GABA Level in Hippocampus of PTZ-Kindled Rats

The hippocampus concentration of GABA was estimated using a quantitative sandwich enzyme linked-immunosorbent assay (ELISA) kit (My BioSource, Milpitas, CA, USA). The protocol of the assay was followed as, described in the kit datasheet. All the samples and standards (50 μL) were incubated with HPR-conjugated reagent (100 μL) for 60 min at 37 °C. After that, all the wells were automatically washed for 4 times using a wash solution. Later, chromogen A (50 μL) and chromogen B (50 μL) were added to each well, and the plate was incubated for 15 min at 37 °C. Afterword, the reaction was stopped by adding (50 μL) of stop solution, and the absorbance was recorded at 570 nm 15 min later. The assay used a standard curve of GABA to calculate the amount of GABA in the samples. GABA concentration was expressed as µmol/mg.

#### 4.5.4. Estimation GLU level in hippocampus of PTZ-kindled rats

The hippocampus concentration of GLU was estimated using a colorimetric assay kit (Biovision Inc, Milpitas, USA). The protocol of the assay was followed, as described in the kit datasheet. All samples and standards with different concentrations were added to the wells in 96-well plate (10 μL), and each well was brought to (50 μL) by assay buffer. The reaction started by adding (100 μL) of the working reagent (GLU assay buffer, GLU developer, and GLU enzyme mix), and the plate was incubated for 30 min at 37 °C. After that, the absorbance was recorded at 450 nm. The assay used a standard curve of Glu to calculate the amount of Glu in the samples. Glu concentration was expressed as nmol/mg.

### 4.6. Measurement of Acetylcholine Esterase Activity

Following previous experimental reports in our laboratories [21,29,145,146], The activity of acetylcholine esterase (AChE) was estimated using colorimetric assay kit (Biovision). Samples (5 µL) were diluted with (45 µL) AChE assay buffer in 96-well plate, then the reaction was initiated by adding (50 µL) reaction mix (AChE assay buffer, AChE probe, AChE substrate and choline oxidase enzyme mix) to each well, after that the 96-well plate was incubated in 37 °C for 20–30 min. Finally, absorbance was read at 570 nm using a micro plate reader.

### 4.7. Measurement of c-Fos protein expression

The day after (24 h) the EPM behavioral test the rats were anesthetized with pentobarbital (40 mg/kg body weight), a cardiac perfusion was carried out using 0.01 M phosphate-buffered saline (PBS) at pH 7.4 and Zamboni solution (Buffered picric acid-formaldehyde). Then the brains were fixed overnight in Zamboni solution. The day after the samples were treated with graded concentrations of ethanol, then xylene and lastly embedded in liquid paraffin. Paraffin blocks were sectioned using microtome. The paraffin-embedded sections were deparaffinized momentarily in a series of alcohol different concentrations then treated with sodium citrate buffer, after that washed in PBS. The sections were treated with blocking agent for 45–60 min, before adding the primary antibody c-Fos mouse monoclonal IgG (1:100, Santa Cruz Biotechnology, Dallas, Texas, USA) for overnight at 4 °C. The primary antibodies against c-Fos were labeled with FITC donkey anti-mouse IgG (Jackson Immuno Research Inc., Dallas, Texas, USA). The sections were covered with glass slides with fluoromount aqueous mounting medium (Sigma-Aldrich), then were observed with a Zeiss fluorescence microscope [147].

## 5. Statistics

Statistical analysis consisted of one-way ANOVA, followed by Tukey’s multiple comparisons test using the software package SPSS 25.0 (IBM Middle East, Dubai, UAE). The *P* values less than 0.05 were considered statistically significant. 

## 6. Conclusions

Taken together, the results of the present study demonstrate that the novel non-imidazole-based H3R antagonist E177 significantly decreased seizure score with concomitant enhancement of memory of PTZ-kindled animals in two different behavioral paradigms. Moreover, E177 mitigated oxidative stress by significantly increasing GSH, CAT, and SOD, and decreasing the abnormal level of MDA. Furthermore, E177 attenuated elevated levels of hippocampal AChE, GLU, and c-Fos protein expression, whereas the decreased hippocampal levels of HA and ACh were modulated. In addition, the E177-provided effects were reversed with the CNS-penetrant H3R agonist RAM, indicating an association between the brain histaminergic neurotransmission and the protective provided by E177. Notably, postulated advantages of a single compound, e.g., H3R antagonist E177, with simultaneous anticonvulsant and memory-enhancing effects over co-administration of two drugs are the straight forward single-compound pharmacokinetics with the lack of supposed drug-drug interactions taking place with combination therapy, and the dose-finding required for co-application of two different drugs (one AED and another memory-enhancing), since the effective doses might be substantially different from the ones applied in case of monotherapy, especially in multifactorial brain disorders like epilepsy. Therefore, the findings suggest the potential of H3R antagonist E177 as adjuvant to currently available AEDs with an added advantage of preventing cognitive impairment, highlighting the histaminergic system as a potential therapeutic target for the therapeutic management of epilepsy with accompanied memory deficits, although further seizure test models with different animal species are still warranted to corroborate and expand these initial data.

## Figures and Tables

**Table 1:** Effect of chronic administration of H3R antagonist E177 on kindling and mortality rates.

Treatment Group	% of Kindling	% of Mortality
SAL	0.00	0.00
PTZ	83.33 ^##^	16.67
E177 (5 mg)	0.00 **	0.00
E177 (10 mg)	0.00 **	0.00
VPA	0.00 **	0.00
E177 (5 mg) + RAM	75.00 ^$$^	8.33
PTZ + RAM	75.00	16.67
SAL + RAM	0.00	0.00

H3R antagonist E177 (5 and 10 mg/kg) and VPA (300 mg/kg) were injected 30–45 min before each PTZ injection. Values are expressed as percentages of the number of animals from each experimental group (*n* = 12). ^##^ *P* < 0.001vs SAL-treated group. ** *P* < 0.001 vs. PTZ-kindled group. ^$$^ *P* < 0.001 vs. H3R antagonist E177 (5 mg)-treated PTZ-kindled rats.
